# Interactive Effects of Immunoglobulin Gamma and Human Leucocyte Antigen Genotypes on Response to Interferon Based Therapy of Hepatitis C Virus

**DOI:** 10.3889/oamjms.2015.046

**Published:** 2015-04-29

**Authors:** Howayda E. Gomaa, Mohamed Mahmoud, Nevine E. Saad, Amal S. Hussein, Somaia Ismail, Eman H. Thabet, Hebatallah Farouk, Dina Kandil, Ahmed Heiba, Wael Hafez

**Affiliations:** 1 *Clinical and Chemical Pathology Department, National Research Centre, Cairo, Egypt*; 2 *Internal Medicine Department, National Research Centre, Cairo, Egypt*; 3 *Environmental and Occupational Medicine Department, National Research Centre, Cairo, Egypt*; 4 *Medical Molecular Genetics Department, National Research Centre, Cairo, Egypt*

**Keywords:** HCV, immunoglobulin gamma, HLA, interferon therapy

## Abstract

**AIM:**

We examined the role that immunoglobulin GM 23 and KM allotypes—genetic markers of γ and κ chains, respectively—play in response to treatment of hepatitis C virus (HCV) infection in Egyptian patients.

**MATERIAL AND METHODS:**

A total of 120 persons who had responded to HCV treatment and 125 with persistent HCV infection were genotyped for the presence of GM23 and KM determinants. HLA –C genotyping was also done.

**RESULTS:**

Association of GM 23+ and KM3 was significantly associated with non response to treatment (P < 0.0001). Individuals who lacked this GM genotype (but were positive for KM1,2 and 3) were likely to respond to treatment (P=0.045). Association of heterozygous GM23 (+/-) with KM 1,2 and 3 or KM3 alone was significantly associated with SVR (P = 0.001) and (P = 0.0001) respectively. Particular combinations of HLA and GM genotypes were associated significantly with the response to HCV treatment. The combination of HLAC2C2 and GM23+ was associated with persistence of infection (P = 0.027) while the association of HLAC2C2 and heterozygous GM23+/- was associated with SVR (P = 0.001). The association of HLAC1C1 and heterozygous GM23+/- was significantly associated with SVR (P = 0.001) and also subjects with HLA C1/C2 and heterozygous GM23+/- were likely to respond to treatment (P = 0.003) while subjects with HLA C1/C2 and GM23+ show tendency to resist to treatment (P = 0.0001).

**CONCLUSION:**

Our results didn’t support a role for KM allotypes, GM23 allotype plays a role in the persistence of HCV infection in the presence or absence of KM1,3. Interaction between certain GM and HLA-C genotypes may favor adequate response to interferon based therapies.

## Introduction

Hepatitis C virus genotype 4 (HCV-4) is the most common variant of HCV in Egypt. Pegylated IFN and ribavirin combination therapy has dramatically improved the treatment response rates, with recent clinical trials showing rates that exceed 60%. There are marked racial differences in rates of clearance of HCV and in responses to interferon-based therapies which may, at least in part, be due to the host genetic differences [1]. Among the host genetic factors, genes of the major histocompatibility complex (HLA) have been the primary focus of studies [2]. The HLA-C genes could influence the outcome of HCV infection by regulating the activity of natural killer (NK) cells. The killer immunoglobulin-like receptors (KIR) on NK cells bind their HLA-C ligands on target cells with differential affinity [3]. In addition, Pandey et al demonstrated the role of interactive effects of GM and HLA genotypes in the outcome of HCV infection.

They also reported involvement of immunoglobulin (Ig) GM and KM allotypes—genetic markers of *gamma* and alpha chains, respectively—in the outcome of HCV infection [1]. Allotypes of IgG proteins are defined by the expression of unique epitopes recognized by unique serologic reagents. Allotypes expressed on the constant region of IgG heavy chain are designated as Gm (Genetic marker) together with the subclass, e.g: G2m and the allotype number, e.g: G2m23. The heavy chains of IgG2 proteins may express the Gm23 allotype that is correlated with the presence of CH2 methionine at residue 282 and CH1 threonine 189.

The human genome has one kappa constant gene. There are three kappa chain allotypes designated Km1, Km2 and Km3 that define three Km alleles: Km1, that correlates with valine153 and leucine 191, Km1,2 that correlates with alanine 153 and leucine 191 and Km3 that correlates with alanine 153 and valine 191 [4].

The HCV core protein has Fc receptor (Fc gamma R)-like properties, which the virus probably exploits to modulate the effector functions of the host immune cells, resulting in the evasion of immunosurveillance [5].

Previous studies revealed that binding of IgG2 of GM23+ allotype to HCV core protein was significantly higher than that of GM23- allotype, IgG2 antibodies directed to the core protein in subjects with this determinant are more likely to have their Fc domains scavenged, thereby reducing their immunological competence to eliminate the virus or circulating nucleocapsids through ADCC and other Fc-mediated effector mechanisms. These studies revealed a higher prevalence of GM23 in subjects with persistent HCV infection compared to those who have cleared the virus [1].

The aim of the present work is to assess the role of GM23 and Km allotypes as well as their interaction with HLA –C type for the prediction of sustained virologic response (SVR) in patients with chronic HCV before initiation of antiviral therapy in order to be able to estimate the potential for treatment success.

## Material and Methods

### Subjects

The study subjects were recruited from several liver centres in Cairo and Giza (Egypt). Many of these patients have received their interferon treatment in Liver Institute of Cairo University. Two groups of cases were participated in this study according to standard criteria:

1-125 cases of chronic HCV patients (non-responder to Interferon therapy).

2-116 cases Sustained virological response (SVR). The patient had a negative HCV-RNA after 6 months of completing 48 weeks (nearly one year) therapy. Patients were treated by either Pegylated or short –acting Interferon along with ribavirin [6].

### Exclusion Criteria

All the cases with HIV, HBV, and shistosoma were excluded. All chronic HCV participants with immune hepatitis or liver complications were excluded.

### Questionnaire and Clinical Examination

A written well informed consent was taken from all the participants. All participants were subjected to full personal and medical history; clinical examination and the status of the liver and spleen were evaluated by ultrasonography. All the cases with history of bilharzial infection and treatment, previous surgery and blood transfusion were excluded. Type of treatment, its duration and response were recorded. The study was approved by the Ethical Committee of the National Research Centre.

### Laboratory Investigations

Estimation of HIV, hepatitis B surface antigen (HBsAg), HBcAb, HBeAg and shistosoma IgG were done by commercially available ELISA kits to exclude the positive cases. ANA, ASMA, AMA and LKM were also measured to exclude immune hepatitis.

The presence of HCV antibodies was determined by third-generation enzyme linked immunosorbent assay (ELISA; CTK-Bioteck-USA), Liver function tests including ALT (alanine transaminase), AST (aspartate transaminase), ALP (alkaline phosphatase) and albumin were assayed using Olympus auto analyser AU400 (Olympus Diagnostica, Japan).

### Detection of HCV-RNA by Real Time PCR and HCV Genotyping

Viral RNA was extracted from patient’s plasma using the QIAamp Viral RNA Kit (Qiagen Hilden, Germany, Cat no. 52904) according to the manufacturer’s protocol. HCV RNA was detected by commercially available Toyobo RNA-direct real time PCR kit on SLAN Real Time PCR Detection System, LG Lifescience, Korea. The HCV genotype was defined by the reverse line probe assay (INNO-LIPA v.1.0, innogenetics, Ghent, Belgium) according to the manufacturer’s instruction.

Determination of immunoglobulin gamma (GM 23) and immunoglobulin kappa (KM) allotypes:

GM23 (valine-to-methionine substitution, a G→A transition in the CH2 region of the γ2 gene) is determined by use of a nested-PCR–restriction fragment length 24 polymorphism (PCR-RFLP) method. In brief, a 915 bp region of the γ 2 gene that incorporates the site for the allelic substitution is amplified by use of primers 5’-AAATGTTGTGTCGAGTGCCC-3’ and 5’-GGCTTGCCGGCCGTGGCAC-3’. The conditions are 4mn at 94°C, 30 cycles (45 s at 94°C, 45 s at 59°C, 1 min 15 s at 72°C), and an extension cycle of 10 s at 72°C. A 197 bp segment is further amplified from this 915 bp fragment, by use of primers 5’-GCACCACCTGTGGCAGGACC-3’ and

5’-TTGAACTGCTCCTCCCGTGG-3’. Digestion of the amplified product by the restriction enzyme *Nla*III run on a7% polyacrylamide (38:2 acrylamide : bisacrylamide) non denaturing gel in TBE buffer for 3 h at 7 V/cm and stained with ethidium bromide, result in the following products corresponding to the following 3 genotypes: GM23+, 90 bp, 63 bp, and 44 bp; GM23−, 134 bp and 63 bp; and GM23+,23−, 134 bp, 90 bp, 63 bp, and 44 bp [7].

*κ*-Chain determinants KM1 and KM3 were characterized by a PCR-RFLP techniqueusing primers 5’-ACTGTGGCTGCACCATCTGTCT-3’ and 5’-TCAGGCTGGAACTGAGGAGCAG-3’. The optimal conditions for PCR are denaturation at 94°C for 1 min, 30 cycles at 68°C for 1 min, and an extension cycle of 1 min at 72°C. Following PCR amplification, digestion of the amplified product (360 bp) by the restriction enzyme *Acc*I result in the following products corresponding to the following 3 genotypes: KM1, 360 bp; KM3, 247 bp and 113 bp; and KM1,3, 360 bp, 247 bp, and 113 bp [8].

Three alleles—KM1, KM1,2, and KM3—segregate at the KM locus. The KM1 allele is extremely rare, and > 98% of subjects positive for KM1 are also positive for KM2; thus, positivity for KM1 includes positivity for both KM1 and KM1,2 alleles.

### HLA Class I Typing

Genomic DNA was extracted from peripheral blood mononuclear cells using the QIAmp DNA minikit (Qiagen) according to the manufacturer’s instructions. HLA-C locus was genotyped by sequence-specific oligonucleotide probe protocols using LABType^®^ SSO Typing kit (ONE LAMBDA, INC): LABType^®^ applies Luminex^®^ technology to the reverse SSO DNA typing method according to manufacturer’s Instructions. Individuals from the two groups (persistent and SVR) were categorized in HLA-C1 and HLA-C2 according to their genotyping data. HLA-C1 and -C2 molecules possess asparagines and lysine at residue 80, respectively.

### Statistical analysis

Statistical analysis were done using computer program SPSS (Statistical Package for the Social Science; SPSS Inc., Chicago, IL, USA) version 18. Data were statistically described in terms of frequencies (number of cases) and percentages. Chi square (χ^2^) test was used for comparing qualitative data. P-values less than 0.05 were considered statistically significant.

## Results

This study included 245 patients with HCV infection; 120 cases of SVR and 125 chronic HCV cases. The age of participants ranged between 20-50 years and 228 of the participated cases were males and 34 were females. The HIV, hepatitis B surface antigen (HBsAg), HBcAb, HBeAg and shistosoma IgG of all the included cases were negative. ANA, ASMA, AMA and LKM of all the included cases were also negative.

The distribution of GM 23 and KM genotypes in relation to response and non response to HCV treatment is given in Table 1.

**Table 1:** Distribution of GM23 and KM genotypes in relation to response and non response to HCV treatment.

Genotype	Persistent HCV N (%)	SVR N (%)
GM 23+	95 (79.2%)	40 (34.4%)
GM 23-	20 (16.7%)	35 (30.1%)
GM 23+/-	5(4.1%)	41 (35.3%)
KM 1,2	2 (1.7%)	0
KM 3	40 (34.8%)	46 (40%)
KM 1,2,3	73 (63.5%)	69(60%)

Persistence n: 125 cases, failed GM23 typing: 5 cases, failed KM typing: 10 cases. SVR n: 120 cases, failed GM23 typing: 4 cases, failed KM typing: 5 cases.

Association of GM 23+ and KM3 was significantly associated with non response to ttt (P < 0.0001). Individuals who lacked GM 23genotype (but were positive for KM1,2 and 3) were likely to respond to treatment (P = 0.045). Association of heterozygous GM23(+/-) with KM 1,2 and 3 or KM3 alone was significantly associated with SVR (P = 0.001) and (P = 0.0001) respectively (Table 2).

**Table 2:** Distribution of combined GM 23and KM 1,2,3 genotypes in relation to response and non response to HCV treatment.

Genotype	Chronic HCV 115 (%)	HCV (SVR) 114 (%)	Chi-square	P-value
GM23+/KM1,2,3	55 (47.8)	29 (25.4)	12.35	P< 0.0001
GM23+/KM1,2	1 (0.86)	0	1.382	0.240
GM23+/KM3	33 (28.7)	11(9.65)	13.38	P< 0.0001
GM23-/KM1,2,3	13 (11.3)	24 (20.9)	4.019	0.045
GM23-/KM1,2	1 (0.86)	0	1.382	0.240
GM23-/KM3	6 (5.2)	10 (8.8)	1.113	0.291
GM23(+/-)/KM1,2,3	5 (4.35)	20 (17.5)	10.251	P=0.001
GM23(+/-)/KM1,2	0	0	Cannot calculated	
GM23(+/-)/KM3	1 (0.86)	20(17.5)	19.11	P< 0.0001

Particular combinations of HLA and GM genotypes were associated significantly with the response to HCV treatment. The combination of HLAC2C2 and GM23+ was associated with persistence of infection (P = 0.027) while the association of HLAC2C2 and heterozygous GM23+/- was associated with SVR (P = 0.001).

The association of HLAC1C1 and heterozygous GM23+/- was significantly associated with SVR (P = 0.001) and also subjects with HLA C1/C2 and heterozygous GM23+/- were likely to respond to treatment (P = 0.003) while subjects with HLA C1/C2 and GM23+ show tendency to resist to treatment (P = 0.0001) (Table 3).

**Table 3:** Distribution of combined HLA- C and IgG(GM 23) genotypes in relation to response and non response to HCV treatment.

Genotype HLA/GM	Chronic HCV 120 (%)	SVR 116 (%)	Chi-square	P-value
C1C1/GM23+	24 (20%)	16 (13.8%)	1.614	0.204
C1C1/GM23-	7 (5.8%)	14 (12.06%)	2.829	0.093
C1C1/GM23+/-	2 (1.7%)	15 (12.9%)	11.19	0.001
C2C2/GM23+	25 (20.8%)	12 (10.3%)	4.908	0.027
C2C2/GM23-	4 (3.3%)	7 (6.0%)	0.968	0.325
C2C2/GM23+/-	0	11 (9.4%)	11.94	0.001
C1C2/GM23+	46 (38.3%)	12 (10.3%)	24.93	<0.001
C1C2/GM23-	9 (7.5%)	14 (12.1%)	1.400	0.237
C1C2/GM23+/-	3 (2.5%)	15 (12.9%)	9.11	0.003

## Discussion

Immunoglobulin (Ig) GM and KM allotypes—hereditary antigenic determinants of IgG heavy chains and kappa-type light chains, respectively—are associated with viral immunological properties and thus are ideal candidate genetic systems for investigations to identify risk-conferring factors in HCV pathogenesis [9].

In the current study, we found that GM23+ allotype was more prevalent in chronic cases than in the SVR group (Table 1). Pandey et al, showed that the prevalence of the GM23-carrying genotype was slightly higher in the group with persistent HCV infection than in the group who had cleared the virus [10] —a finding that is consistent with the observation that GM23-carrying IgG2 has a higher affinity for binding to the core protein than does its allelic counterpart. Previous studies have established that the recombinant HCV core protein does not bind to the Fab fragments of “nonimmune” IgG, the binding between IgG2 and the HCV core protein involve the Fc region of the IgG2 molecules. The differential binding of the HCV core protein to the GM23-disparate IgG2 proteins influence the strategies employed by this virus to evade host immunosurveillance. Fc *gamma*R-like HCV core protein binds anti-core antibodies by “bipolar bridging.” In this model, the Fab part of the antibody molecule (paratope) binds to its antigenic target (epitope), whereas the Fc part of the antibody binds to the Fc gamma R-like binding site on the viral protein [5]. This binding may offer survival advantage to the virus by sterically hindering the access of Fc R-expressing effector cells to HCV-infected cells. The HCV core protein may scavenge the Fc domains of anti-core antibodies after the binding of their paratopes to their antigenic target, thereby interfering with the effector functions—such as antibody-dependent cellular cytotoxicity (ADCC)—mediated by the Fc domain of the bound antibody. Although antibody responses to HCV proteins are predominantly of IgG1 and IgG3 subclasses, antibodies of the IgG2 isotype have also been reported [11].

In our study, we didn’t found an interactive effect of GM23 allotype and KM allotypes, analysis of the results in Table 2 revealed that individuals who lacked or were heterozygous for GM23 allotype and were positive for KM1,3 were likely to respond to treatment and clear the infection but in the presence of homozygous GM23 (and in the presence of KM1,3), subjects were less likely to respond to treatment. Our results didn’t support a role for KM allotypes. In a study done by Pandey et al (2004), he reported that subjects with GM 1,17 5,13 and KM 1,3 phenotypes were over three times (odds ratio [OR], 3.57; 95% confidence interval [CI], 1.44 to 8.87) as likely to clear the infection as the subjects who lacked these phenotypes. This GM phenotype had a similar association with clearance in the absence of KM 3 (OR, 2.75; 95% CI, 1.21 to 6.23). The presence of GM 1, 3, 17, 23, 5, 13 phenotype (in the absence of KM 3) was associated with persistence (OR, 0.21; 95% CI, 0.06 to 0.77), while its absence (in the presence of KM 1,3) was associated with the clearance of infection [9]. Therefore, in accordance with our results GM23 allotype plays a role in the persistence of HCV infection in the presence or absence of KM3.

In the present study, we found interactive effects of GM and HLA genotypes to respond or not to interferon based therapies.

Our results show that subjects with HLAC2C2 or HLAC1C2 and homozygous GM23+ were likely to resist to treatment (P = 0.027 and P = 0.0001 respectively), while subjects with HLAC1C1 or HLAC1C2 and heterozygous GM23+/- have tendency to respond to treatment (P = 0.001 and P = 0.003 respectively).

The HLA-C genes investigated in this study could influence the outcome of HCV infection by regulating the activity of natural killer (NK) cells, key players in anti-viral immunity. Particular allelic combinations of KIR and HLA-C genes have been reported to be associated with the resolution of HCV infection in one study [3], and confirmed in our previous study [12]). In the study done by Khakoo *et al*, on Caucasian and African Americans, the frequency of HLA-C1homozygosity was higher in individuals with resolved infections. They suggested that HLA-C1homozygosity might have a protective effect on HCV infected hosts, because of the capacity of these molecules to present antigens that have stronger affinities for cytotoxic T cells [3].

Previous studies showed that binding of IgG2 of GM23(+) allotype to HCV core protein was significantly higher than that of GM23(-) allotype, IgG2 antibodies directed to the core protein subjects with this determinant (GM23) are more likely to have their Fc domains scavenged, thereby reducing their immunological competence to eliminate the virus or circulating nucleocapsids through ADCC and other Fc-mediated effector mechanisms [5]. Therefore, the interactive effect between both HLA-C groups and the presence or absence of GM 23allotype observed here could be explained by the additive effect of NK-dependent ADCC against HCV-infected cells by the HLA-C molecules and the influence of GM23 on the virus to evade host immune response. Specific combinations of HLA-C and GM genes may favour adequate response to interferon based therapy.
